# One-Pot Synthesis of Glucose-Derived Carbon Coated Ni_3_S_2_ Nanowires as a Battery-Type Electrode for High Performance Supercapacitors

**DOI:** 10.3390/nano11030678

**Published:** 2021-03-09

**Authors:** Zhongkai Wu, Haifu Huang, Wenhui Xiong, Shiming Yang, Huanhuan Huang, Yaohui Zou, Weiping Zhou, Zhenzhi Cheng, Jun Wang, Guangsheng Luo

**Affiliations:** 1 School of Materials Science and Engineering, Nanchang University, Nanchang 330031, China; 401329118005@email.ncu.edu.cn (Z.W.); 411314019080@email.ncu.edu.cn (W.X.); 411314019084@email.ncu.edu.cn (S.Y.); 411314018061@email.ncu.edu.cn (H.H.); 411314019105@email.ncu.edu.cn (Y.Z.); wpzhou@ncu.edu.cn (W.Z.); chengzz@ncu.edu.cn (Z.C.); 2 Nanjing National Laboratory of Solid State Microstructures, School of Physics, Nanjing University, Nanjing 210093, China; 3 School of Resource, Environmental and Chemical Engineering, Nanchang University, Nanchang 330031, China; 4 Guangxi Novel Battery Materials Research Center of Engineering Technology, Center on Nanoenergy Research, School of Physics Science and Technology, Guangxi University, Nanning 530004, China; haifuh@163.com (H.H.); jwang7@ncu.edu.cn (J.W.); 5 State Key Laboratory of Crystal Materials, Shandong University, Jinan 250100, China

**Keywords:** Ni_3_S_2_, rod-like structure, one-step hydrothermal method, carbon coating, supercapacitor

## Abstract

We report a novel Ni_3_S_2_ carbon coated (denoted as NCC) rod-like structure prepared by a facile one-pot hydrothermal method and employ it as a binder free electrode in supercapacitor. We coated carbon with glucose as carbon source on the surface of samples and investigated the suitable glucose concentration. The as-obtained NCC rod-like structure demonstrated great performance with a huge specific capacity of 657 C g^−1^ at 1 A g^−1^, preeminent rate capability of 87.7% retention, the current density varying to 10 A g^−1^, and great cycling stability of 76.7% of its original value through 3500 cycles, which is superior to the properties of bare Ni_3_S_2_. The result presents a facile, general, viable strategy to constructing a high-performance material for the supercapacitor applications.

## 1. Introduction

With the consumption of fossil energy and the increase of the energy demand, high-performance supercapacitors have attached researchers attention on the account of their high specific power, fast charging-discharging ability, and excellent stability [1,2,3,4]. Based on the different energy storage mechanisms, supercapacitors can be divided into two kinds: double-layer capacitors and faradaic pseudocapacitors, owing to their capability of a rapid and effective charging-discharging redox process occurring on the electrode surface, whereby pseudocapacitors could present better specific capacity and specific energy than double-layer capacitors [5,6]. Moreover, the electronegativity of transition metal sulfides is lower than that of transition metal oxides, especially in double metal cobalt nickel sulfide, their electrochemical activity and conductivity was obviously higher than that of oxide/hydroxyl [7]. Moreover, its mechanical and thermal stability is also better than oxide/hydroxyl. In addition, due to the effective combination and various valence state of nickel sulfide, they have more active sites than the metal oxide during the REDOX reaction, thus greatly improving the electrochemical activity [8]. As a result, compared with a traditional metal oxide electrode, metal sulfides like NiS [9], CoMoS_4_ [10], and Co_8_S_9_ [11] have gained intensive attention of scientists due to their better electrical conductivity, good rate performance, and outstanding specific capacity. Meanwhile, the high cost and toxicity of Ni/Co-based metal limits its appliance in supercapacitors, the preparation of a metal sulfide materials with low pollution, low cost and high performance has aroused insensitive interest of many scientists [12,13,14,15].

Designing a rational and controllable morphology is also critical to improve the electrochemical performance. Many efforts has been made to construct a novel nickel sulfide nanoarchitecture [16,17,18], and these papers evince that nanoarchitecture such as particles [19], wires [20], rods [21,22], flakes [23,24], and flowers [25] have great advantages in promoting electrochemical performance of electrode materials. For example, Ji [26] reported a simple in-situ Ni_3_S_2_ thin film by a facile hydrothermal method, the as-obtained sample exhibits outstanding specific capacitance of 2230 F g^−1^, good rate property and high capacitance retention of 91% of its initial value. Shen [27] reported a low-temperature sulfurization by the assistance of oxidant K_2_S_2_O_8_ to prepare a large number of porous Ni_3_S_2_ doughnuts, the composite demonstrates high specific capacitance of 2519.5 mF g^−1^, good rate performance of 68% retention over the current density varying from 1 mA cm^2^ to 20 mA cm^2^. Zhang [28] reported a dendritic structure via one-pot hydrothermal process, the Ni_3_S_2_ dendrites show high specific capacitance of 710.4 F g^−1^ at the current density of 2 A g^−1^ and great rate performance and stability. Above all, Ni_3_S_2_ is a controllable and promising electrode material [29]. Notwithstanding the achievements, transition metal sulfide also has the defects of poor rate performance and low cycling life, in order to solve these difficulties, an alternative strategy to improve the poor intrinsic conductivity of transition metal sulfide is to combine it with carbon material like graphene, carbon nanotubes. The expected product can obtain better rate performance and cycle stability due to the electrical double-layer capacitance of carbon materials and the factors above: (i) Carbon-based materials can improve the conductivity and cyclic stability of composites, and overcome the problem of poor electrical conductivity of metal sulfide materials; (ii) the direct contact area between the active material particles and the electrolyte is decreased, thus reducing the formation of passivation layer on the surface of the active material particles; (iii) the elastic phase in the carbon nanocomposite can effectively absorb the volume change caused by the reaction between the active material and the electrolyte, and increase the structural stability of the material; (iv) in the process of charge and discharge, the presence of carbon material can effectively inhibit the agglomeration of active material particles.

To sum up, compared to other metal sulfides and oxide, we think nickel sulfide is a promising material to construct supercapacitor electrodes. Meanwhile, fabricating an electrode material with unique morphology and high specific surface area, and using carbon materials to form composites is a key factor to improve the performance of supercapacitors. In view of this, we developed an idea of directly growing carbon layer on the nickel sulfide surface, using glucose as carbon source, Ni_3_S_2_ would take part in the redox reaction to provide pseudocapacitive character and the carbon coating will contribute towards EDLC along with controlling the agglomeration of the overall structure. Furthermore, such method have the following advantages: First, the glucose can be added directly in the hydrothermal process, so that the growth of nickel sulfide and the coating of carbon layer are carried out in one step, which simplifying the reacting procedure. Second, The as-obtained carbon layer can be directly coated on the surface of nickel sulfide through the hydrothermal reaction process, and the composite material is expected to have a good bonding property. Third, compared to carbon nanotubes, graphene and other carbon materials, using glucose as a carbon source has a lower cost.

In this line, we propose a carbon coated Ni_3_S_2_ wires structure by a one-pot hydrothermal method which Ni foam was directly used as substrate and Ni source; TAA(thioacetamide) as S source and glucose as C source. Composites were uniformly aligned on the Ni foam substrate, and the mass of coated carbon can well be controlled. Based on the synergistic effect between carbon and sulfide, as-prepared sample exhibits high specific capacity, good rate performance and outstanding stability. Furthermore, a hybrid supercapacitor using NCC as positive electrode and active carbon as negative electrode was fabricated, the device exhibits excellent electrochemical properties.

## 2. Experimental Section

### 2.1. The Preparation of Bare Ni_3_S_2_ Rod-Like Structure

In a typical synthesis procedure, a clean Ni foam was prepared advanced by rinsing through 3 M HCl, acetone and distilled water, respectively to clean up contamination on the electrode surface, 10 mmol CH_4_N_2_O and 8 mmol TAA(thioacetamide) was subsequently dissolved into 50 mL DMF (Dimethyl formamide) and stirring for 20 min to acquire transparent solution, the cleaned Ni foam and transparent solution after stirring were shifted into a 100 mL Teflflon-lined stainless steel autoclave, and keeping the temperature at 160 ℃ for 8 h. Finally, the product was naturally cooled down in room temperature to obtain bare Ni_3_S_2_ rod-like structure.

### 2.2. The Preparation of NCC-0.5/1/1.5

NCC-0.5/1/1.5 was prepared via a similar procedure. A piece of nickel foam with a size of 1 cm^2^ was treated by a 3 M HCl, acetone and distilled water to remove the contamination on the surface, subsequently, 10 mmol CH_4_N_2_O, 8 mmol TAA along with a certain quantity of glucose was added into 50 ml DMF solution under constantly stirring for 10 min (with the glucose mass of 0.5 g; 1 g and 1.5 g, corresponding to the concentration of 10 L^−1^, 20 L^−1^ and 30 L^−1^). Then, the mixture was transferred into 100 mL Teflon-lined stainless-steel autoclave, and keeping the temperature at 160 ℃ for 8 h. After cooling down to room temperature, the sample was washed and dried for 12 h to obtain Ni_3_S_2_ carbon coated structure (noted as NCC-0.5; NCC-1 and NCC-1.5, respectively).

### 2.3. Characterization of Samples

X-ray diffraction (XRD) was performed on a X-ray diffractometer (PANalytical-Empyrean, Panalytical, Amsterdam, The Netherlands) with Cu Ka radiation (λ = 0.15406 nm) at a scanning speed of 2 min^−1^. Scanning Electron Microscope (SEM,JSM5800, FEI, Hillsboro, OR, USA) and transmission electron microscopy (TEM, JEM2010, JEOL, Tokyo, Japan) was employed to investigate the surface microtopography of the material. Raman spectrum was characterized via Laser Raman Spectrometer(Autosor-iQ, Jobin Yvon, Paris, France). Dispersive spectrometry (EDS) were executed via Oxford INCA model 7421 energy dispersion spectrometer(AZtec X-Max 80, Oxford Instrument, Oxford, UK). Specific area of the sample was confirmed through Brunauer-Emmett-Teller (BET) approach(Autosor-iQ, Quantanchrome instruments, Boynton, the United States).

### 2.4. Electrochemical Tests

All the electrochemical tests was proceed by three-electrode cell workstation (CHI 660E, Chenhua, Shanghai) in 3 M KOH aqueous solution while platinum sheet was served as counter electrode, as-obtained nickel sulfide was as positive electrode material and saturated calomel electrode (SCE) was as reference electrode. CV (cyclic voltammetry), GCD (galvanostatic charge-discharge) and EIS (electrochemical impedance spectroscopy) and cycling stability tests was executed to investigate the electrochemical properties. Mass specific capacitance (*Cm*) can be calculated by the following equation [30]:

*Cm = I × t/m × U* (1)

where *I*(A) represent current conducted on electrode materials, *t*(s) stands for the discharging time, *m*(g) signifies active mass of the electrode, and *U*(V) refers to the discharge potential window.

HSC (hybrid supercapacitor) device was fabricated, NCC-1 as a battery-type positive electrode while active carbon (AC) was as negative electrode, samples was cut into round shape with the radius of 0.5 cm in order to fit the cell, AC and polyvinylidene fluoride (PVDF) was mixed with of mass ratio of 9:1, then uniformly coated on Ni foam to obtain a AC negative electrode. Finally, the positive and negative electrode were put into the button cell using 1 M KOH, filter as electrolyte, and a separator for assembling the device. The specific energy and specific power were calculated via the formula:

E = 0.5 × *C_m_* × *U*^2^/3.6 (2)

P = 3600 × E/*t* (3)

where *U*(V) present voltage window, *C_m_*(C/g) imply the specific capacity, *t*(S) signifies the discharging time. 

## 3. Results and Discussion

**Figure 1:**
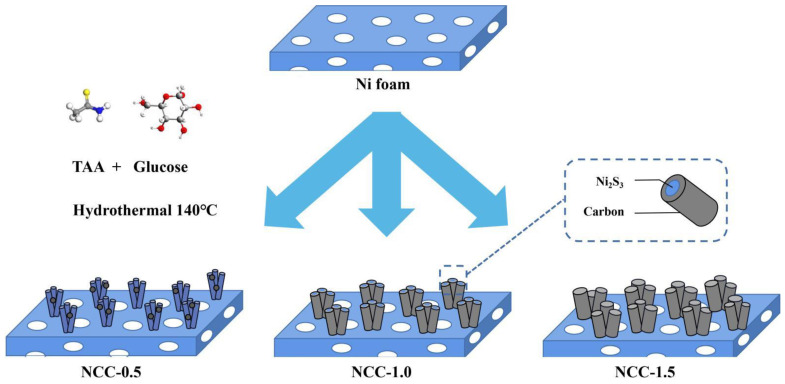
Schematic illustration of the synthesis of Ni_3_S_2_ on Ni foam based to different temperature ranges.

The preparation of NCC-0.5/1/1.5 rod-like structure was shown in Figure 1, first, the Ni foam was directly used as the Ni source and substrate, TAA (thioacetamide) was added as S source, glucose was as C source. Finally, NCC was grown on the Ni surface though hydrothermal sulfidization and self carbon-coating process, Ni_3_S_2_ nanowires was first synthesized from the hydrothermal process, then, the carbon material was wrapped on the Ni_3_S_2_ nanowires, forming a well-defined core-shell heterostructure. The growing process of nickel sulfide and the carbon coating step are integrated within a facile one-pot hydrothermal method, simplifying the reaction steps without calcination and other subsequent processing.

**Figure 2:**
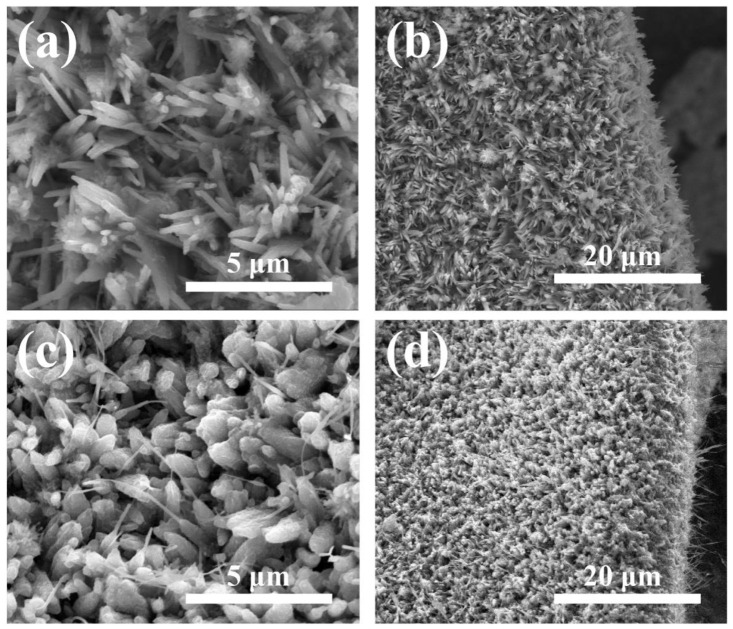
SEM images of the Ni_3_S_2_ nanorods before and after carbon coating in different magnification: (**a**,**b**) bare Ni_3_S_2_ nanorods; (**c**,**d**) Ni_3_S_2_ carbon coating nanorods (NCC-1).

SEM images of NCC-0.5/1/1.5 and bare Ni_3_S_2_ in different magnification was shown on Figure 2a–d, it can be seen that, bare Ni_3_S_2_ nanowires were uniformly grown on the Ni foam, which could act as scaffolds for the subsequent growth of carbon shell. As-obtained Ni_3_S_2_ sample directly growing on the substrate demonstrates rod-like structure with the width of nearly 500 nm. While the high magnification image shows the controlled growth of NiS2 in one plane. Figure 2c,d exhibits carbon coated Ni_3_S_2_ rod-like structure, which can be seen that, through one-pot hydrothermal process, the carbon layer was uniformly wrapped on the sulfide surface. the rods are well-aligned and remain the similar morphology before and after carbon coating. The introduction of carbon was found to be helpful in controlling the grain growth of overall structure suggesting that there was no irregular growth, and all the structures were of uniform size.

**Figure 3:**
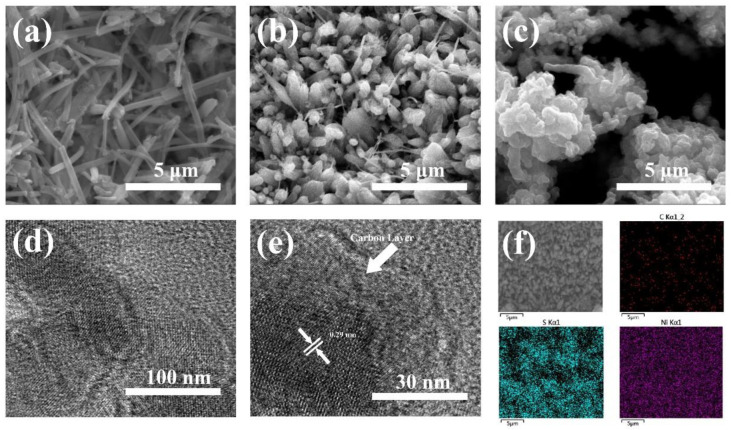
SEM images of (**a**) NCC-0.5, (**b**) NCC-1 and (**c**) NCC-1.5; HRTEM images of NCC-1 (**d**,**e**); EDS mapping of NCC-1 (**f**).

To investigate the effect of glucose concentration to carbon layer, the morphology of NCC structure with different mass ratio of glucose is further analyzed in Figure 3a–c. By regulating the concentration of the glucose, we obtained samples coated with different amount of carbon, as to Figure 3b, it is identified that the shape and size of the nanowires are not much altered even after carbon coating. When the glucose precursor concentration is relatively low (10 g L^−1^), glucose pellets were uniformly distributed on the surface of nanowires (Figure 3a). If the concentration reaches a relatively high level (30 g L^−1^), the carbon layer not only coated the nanowires, but also generated excessive carbon on the surface of the sample, resulting in a change in the morphology of the sample (Figure 3c). The carbon layer can be uniformly coated on the surface of the as-prepared sample (Figure 3b) only when the glucose concentration is suitable (20 g L^−1^). Moreover, transmission electron microscope (TEM) tests were also employed to analyze the internal structure of the materials, as can be seen from Figure 3d,e, a single Ni_3_S_2_@Carbon nanowires was prepared, Ni_3_S_2_ encapsulated by carbon layer have the average diameter of 80 nm, the thickness of outer amorphous carbon layer is approximately 20 nm. In addition, HRTEM image reveals well-resolved lattice fringes of 0.29 nm, which correspond to the typical (110) crystal planes of Ni_3_S_2_.

The energy dispersive spectrometer test (EDS) for Ni, S, and C were also conducted to explore the elemental distribution of the material. From Figure 3f, the material consisted of these three elements above. Meanwhile, the atomic percentage of the element Ni and S is 45.4:19.6, which is very close to 3:2, indicating we have successfully prepared Ni_3_S_2_@Carbon material.

**Figure 4:**
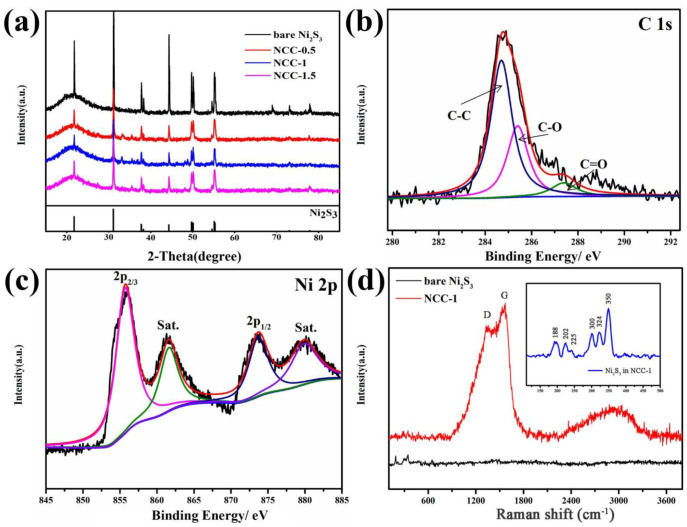
XRD patterns of NCC-0.5, NCC-1 and NCC-1.5 (**a**); C 1s spectrum (**b**); XPS patterns of (**c**) C1s and (**d**) Ni2p of NCC-1; Raman spectrum of bare Ni_2_S_3_ and NCC-1.

Figure 4a shows the XRD patterns of NCC-0.5, NCC-1 and NCC-1.5, for the XRD pattern of NCC, the diffraction peaks at 23.4°, 33.5°, 47.9°, 53.8°, 54.2°, and 75.4° can be attributed to the (101), (110), (202), (113), (211), and (131) planes of hexagonal Ni_3_S_2_ (JCPDS 44-1418), respectively, indicating the presence of crystalline Ni_3_S_2_. However, the presence of carbon was not clearly detected on image, which could possibly because of too much weak to obvious. Other characters such as the SEM, EDS mapping, and XPS measurements confirmed the presence of carbon. Moreover, it is known that XRD diffraction peak is corresponding to a certain crystal surface, higher intensity indicating the abundant array of atoms on the material surface, and larger number of surface atoms lead to higher crystallization. As a result, as for the NCC-0.5, NCC-1 and NCC-1.5, there is no significant difference in peak intensity, but bare Ni_3_S_2_ has the highest intensity, which is because the combination of nickel sulfide and amorphous carbon reduces the crystallinity of the material, leading to lower intensity of NCC-0.5/1/1.5.

XPS was undertaken to analyze the valence state of the elements. Figure 4b presents the C 1s spectrum, exhibits three-type peaks at 284.7, 285.4, and 287.4, which are respectively attributable to C–C/C–O, C=O bonds [31]. From Figure 4c, The spectrum of N 2p is divided into four components peaks at 855.8, 861.7, 873.7 and 880 eV, two main peaks and two satellite peaks can be observed in Ni region, the two main peaks at 855.7 and 873.7 eV are belong to 2p 3/2 and 2p1/2, respectively, while two peaks located at 861.7 eV and 880 eV are belong to satellite peaks.

Raman spectra were recorded for bare and carbon-coated Ni_3_S_2_(NCC-1) and are presented in Figure 4d. On the account of strong intensity of carbon peak, in order to improve the measurement accuracy, we set a small Raman shift range of 100–500 cm^−1^ to NCC-1. The small image inserted in the Figure 4d reveals an obvious Ni_3_S_2_ peak, demonstrating the presence of nickle sulfide. Meanwhile, obvious difference between bare and NCC-1 can also be seen, both patterns exhibited the characteristic bands at 187, 201, 222, 303, 324, 350 cm^−1^. The observed Raman bands are consistent with the previous reports on Ni_3_S_2_ [32,33,34]. In addition to the above characteristic bands, NCC-1 clearly reveals the existence of carbon with its two characteristic bands at 1330 and 1513 cm^−1^. The band at 1513 cm^−1^ corresponds to the G band, whereas the broad band (D) at 1330 cm^−1^ is related to the disordered carbon. The peak intensity ratio of D and G bands (ID/IG) provides information about the degree of crystallinity of the carbon covered on the Ni_3_S_2_. In the present case, the ID/IG ratio is 0.83, depicting a typical amorphous carbon structure, which agrees well with the XRD and TEM measurements [35].

**Figure 5:**
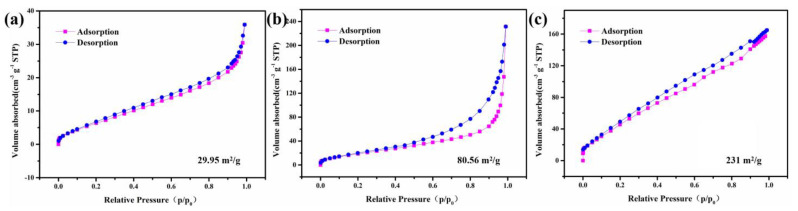
N_2_ adsorption–desorption isotherm of (**a**) NCC-1.5; (**b**) NCC-0.5; (**c**) NCC-1.

N_2_ adsorption–desorption isotherm of NCC-1.5, NCC-0.5 and NCC-1 are also investigated via Figure 5, it can be seen that the specific area of NCC-1.5, NCC-0.5 and NCC-1 reached 29.95 m^2^/g, 80.56 m^2^/g, and 231 m^2^/g, respectively, indicating that NCC-1 possess largest specific area. which are consistent with the microscopical observation of as-prepared NCC-1. Note that the large specific surface areas brought by the carbon coating will undoubtedly shorten the ion diffusion paths and enhance the utilization of active materials, which can contribute to an improved pseudocapacitive performance.

**Figure 6:**
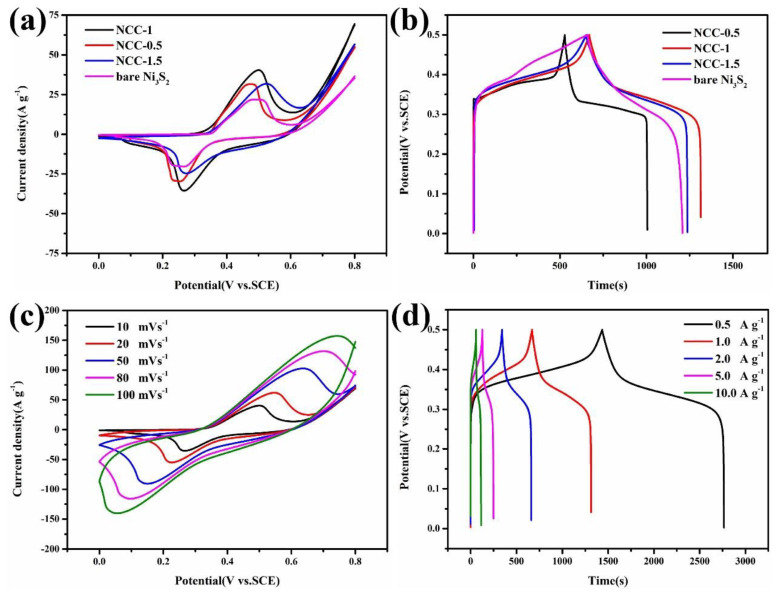
(**a**) Cyclic voltammetry curves and (**b**) charge-discharge curves of bare Ni_3_S_2_; NCC-0.5, NCC-1.5 and NCC-1 at the scan rate of 10 mV s^−1^ and in the potential window of 0.5 v and the current density of 1 A g^−1^; (**c**) Cyclic voltammetry curves and (**d**) charge-discharge curves of NCC-1.0 under various scan rate and current density.

**Figure 7:**
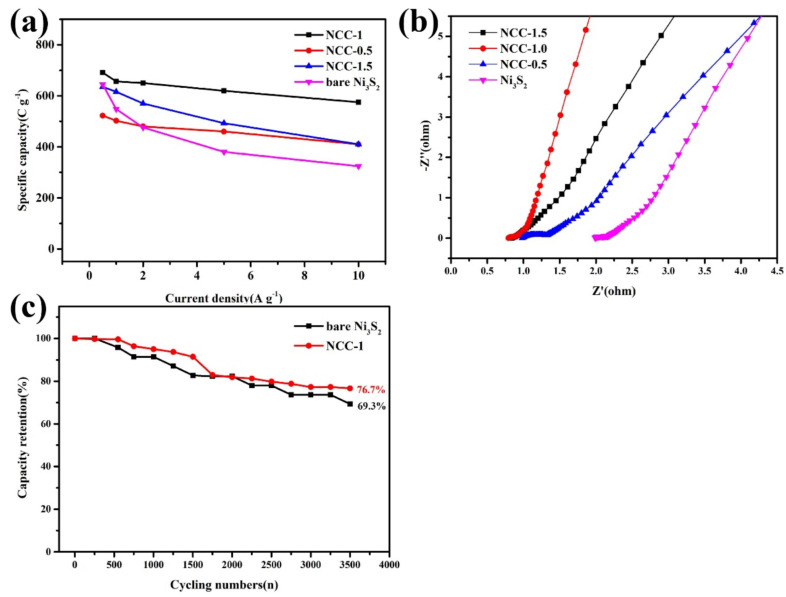
(**a**) Specific capacity at different current density and (**b**) Nyquist plots of EIS of Ni_3_S_2_; NCC-1.5, NCC-1.0 and NCC-0.5; (**c**) Comparison of cycling stability test between bare Ni_3_S_2_ and NCC-1.

The electromechanical test of the obtained sample is shown in Figure 6 and Figure 7. Figure 6 illustrates, CV (Cyclic voltammetry) curves at the scan rate of 10 mV s^−1^ and GCD (galvanostatic charge-discharge) curves under the current density of 1 A g^−1^ of NCC-1.5, NCC-0.5, and NCC-1. From the images of CV curves, the as-obtained sample shows redox peaks evidencing the typical Faradaic pseudocapacitance behavior. Moreover, covering area of the NCC-1 is larger than that of NCC-1.5 and NCC-0.5, revealing a better electrochemical performance of NCC-1. The observed redox peaks are assumed to be reversible electron transfer process occurred on the surface of the Ni_3_S_2_ electrodes by the equation:

Ni_3_S_2_ + 3OH^−^ = Ni_3_S_2_(OH) _3_ + 3 e^−^ (4)

Corresponding to the CV test, GCD curves of three electrodes are shown in Figure 6b, discharging time of NCC-1 reaching 657s, which is longer than that of NCC-0.5 (503s), NCC-1.5 (548 s) and bare Ni_3_S_2_ (616 s), resulting the capacity of 657 C g^−1^, 503 C g^−1^ 548 C g^−1^ and 616 C g^−1^, respectively (calculation method is according to the Equation (1)). CV curves at various scan rate and GCD curves with different current density of sample NCC-1 was demonstrated in Figure 6c,d), it can be seen that as the scan rate increased, the cathodic peak moves to negative direction and the anodic peak position moves to positive direction while CV curves remain similar shape, which revealing excellent rate performance of NCC-1. From the Figure 6d, GCD curves of NCC-1 with various current density was investigated, the obvious platform exhibited on the image evidencing the representative battery-type behavior which derived from the redox reaction occurred on the interface. In the current density of 1 A g^−1^, NCC-1 present discharging time of 657 s, corresponding capacitor of 657 C g^−1^ with 1 mg of mass of active martial. Even at the high density of 10 A g^−1^, the capacity still shows capacity retention of 83.2% to its value in 0.5 A g^−1^.

For comparison, plot diagram of NCC-0.5, NCC-1.5 and NCC-1 was further explored in Figure 7a, The as-obtained samples demonstrated similar capacity retention of 78.4% (NCC-0.5), 64.7% (NCC-1.5) 83.2% (NCC-1) and 50.2% (bare Ni_3_S_2_) (from 0.5 A g^−1^ to 10 A g^−1^), such great rate ability can be attributed to the unique nanorods arrays structure directly growing on the Ni substrate without the addition of binder or conductive material and the improvement of electrical conductivity brought by carbon coating. Nyquist plots of EIS was carried out in Figure 7b to investigating electrochemical behavior, as can be seen, intercept at the real axis (Z0) represent the combined resistance (Rs) between electrode and electrolyte, NCC-1 and NCC-1.5 present smallest intercept, which indicates good electrical conductivity. Furthermore, the plot displays a nearly vertical line in the low frequency region, which is the evidence of relatively low ionic diffusion resistances of the NCC-1. The semicircle represents charge transfer resistance (Rct) caused by Faradic reactions, and the smaller diameter size of NCC-1 suggesting lower combined resistance than that of NCC-1.5 and NCC-0.5. Meanwhile, cycling stability test was conducted via GCD measurement within a constant current density of 5 A g^−1^ over 3500 cycles, the result present the decline of the specific capacitance with the retention of 76.7% of its original value, demonstrating enhanced cycling ability than bare Ni_3_S_2_. The improvement of stability is due to the improvement of electronic conduction characteristics of the composites by carbon-sulfide materials, the carbon layer on the surface of the sample protects the inner layer, which weakens and limits the damage caused by expansion stress to the material structure during the charge and discharge period.

Table 1 compares the electrochemical properties and synthesizing method for different Ni_3_S_2_@carbon composites. The results reveal the enhanced specific capacity, best rate performance, and excellent cycling stability of as-obtained NCC-1. The improvement to the properties can be attributed to the shorter diffusion path and synergistic effect between materials.

**Figure 8:**
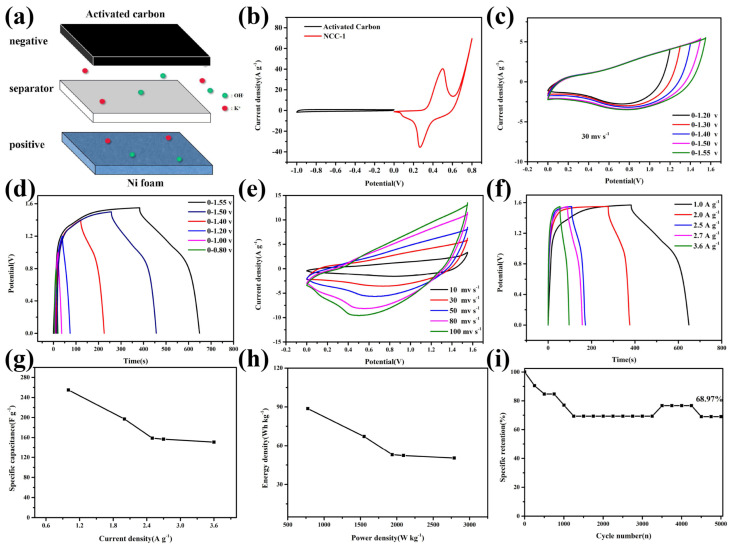
(**a**) Schematic diagram of the HSC device, (**b**) CV comparison curves of NCC-1 and AC electrodes, (**c**) CV curves and (**d**) GCD curves of NCC-1 in the potential range of 0.8–1.55 V at a scan rate of 50 mV s^−1^ at the current density of 1.0 A g^−1^, (**e**) CV curves and (**f**) GCD curves of NCC-1 device at various scan rates and current densities at the voltage window of 1.55 V, (**g**) Specific capacitance of the NCC-1 device in different current densities, (**h**) Ragone plot of the NCC-1 HSC device, (**i**) Cycling stability test of NCC-1 device.

To analyze the practical application of NCC-1 as battery-type electrode. Figure 8a reveal the schematic diagram for the assembling process, HSC (NCC-1//AC) device was fabricated, consisting of NCC-1 as a positive electrode while AC@NF as negative electrode and filter as separator in KOH electrolyte. Figure 8b depicts a comparison for the CV curves of NCC-1 and AC under the scan rate of 10 mv s^−1^, from the image, CV curves of AC demonstrates a rectangle shape, which implying the typical behavior of EDLC. Moreover, NCC-1 and AC electrode present disparate potential window of −1–0 V and 0–0.8 V, respectively. Different voltage windows of positive and negative electrode resulting a wider range of operating voltages, thus improving the specific energy of the device.

In order to investigate maximal operating potential range, CV and GCD measurement of the device at various potential window was exhibits in Figure 8c,d, CV and GCD test was carried at the scan rate/current density of 30 mv s^−1^ and 1 A g^−1^, respectively. It can be seen that the potential window gradually increased, general shape of the curves remain unchanged, indicating the maximum potential of 1.55 V. The electrochemical properties of HSC device was further verified through CV and GCD measurement at different scan rates and current densities, from Figure 8e,f, as the scan rate elevates, CV curves still retain similar form, GCD curves exhibits symmetrical shape and an obvious plateau, reach a potential of 1.55 V, implying a faradic battery-type behavior. Specific capacitance of the device was plotted in Figure 8g, calculated at 255 F g^−1^ at 1 A g^−1^ and maintained 58.8% of its initial capacitance even the current density elevated to 3.6 A g^−1^ (the sudden capacity increasing might be caused by the shedding of carbon layer during charge-discharge process, exposing more capacitive sulfide material). Figure 8h depicts the Ragone plot of device’s specific energy and specific power, as-obtained device exhibits highest specific energy of 88.7 Wh kg^−1^ at the specific power of 775 W kg^−1^ and still kept an specific energy of 50.33 Wh kg^−1^ even when the specific power reached up to 2790 W kg^−1^. Cycling stability test was performed after 5000 cycles and the device demonstrates great stability with a capacitance retention ratio of 68.97%.

## 4. Conclusions

In this work, we have successfully prepared a rod-like Ni_3_S_2_@carbon structure by the one-pot hydrothermal method. A different morphology was controlled by modulating various concentration of glucose and the as-prepared Ni_3_S_2_ carbon coating rod arrays exhibits best electrochemical property when the glucose concentration is 20 g L^−1^, with the high specific capacity of 657 C g^−1^ at the current density of 1 A g^−1^, and with excellent stability of 76.7% retention after 3500 cycles. Moreover, an HSC device was fabricated and the as-obtained device demonstrated a high specific energy of 88.7 Wh kg^−1^ at the specific power of 775 W kg^−1^. The facile strategy for directly using Ni foam as substrate and Ni source with a simple one-step hydrothermal method can provide a protocol for the fabrication of high-performance supercapacitors. 

## Figures and Tables

**Table 1:** Method of synthesis, Specific capacity, Rate performance and Capacity retention of NCC-1 and other Ni_3_S_2_@Carbon materials reported in previous literatures.

Materials	Method of Synthesis	Specific Capacity (C g^−1^)/Current Density (A g^−1^)	Rate Performance from 1 to 10 A g^−1^	Capacity Retention/Cycles
NiS@N-doped carbon sphere [36]	Multistep Transformation method	517.5 C g^−1^/1 A g^−1^	78%	76%/4000
Ni_3_S_2_@CNF [37]	Hydrothermal	380.6 C g^−1^/2 A g^−1^	83%	88%/1500
Ni_3_S_2_@rGO [38]	Vacuum filtration method	477.37 C g^−1^/2 A g^−1^	76%	88%/5000
Ni_3_S_2_@Graphene [39]	CVD method	372 C g^−1^/1 mv s^−1^	69.3%	92.3%/5000
Ni_3_S_2_@CNT [40]	Hydrothermal	360 C g^−1^/3.2 A g^−1^	58.6%	80%/1000
Ni_3_S_2_@PAN [41]	Electrochemical deposition	440 C g^−1^/1 A g^−1^	78.73%	93.14%/2500
NCC-1 (this work)	Hydrothermal	657 C g^−^^1^/1 A g^−1^	87.7%	76.7%/3500

## Data Availability

3rd Party Data Restrictions apply to the availability of these data. Data was obtained from Nanchang University and are available from the authors with the permission of Nanchang University.
